# A Systematic Review on the Efficacy and Safety of Selective Serotonin Reuptake Inhibitors in Gastrointestinal Motility Disorders: More Control, Less Risk

**DOI:** 10.7759/cureus.27691

**Published:** 2022-08-04

**Authors:** Maitri V Acharekar, Sara E Guerrero Saldivia, Sumedha Unnikrishnan, Yeny Y Chavarria, Adebisi O Akindele, Ana P Jalkh, Aziza K Eastmond, Chaitra Shetty, Syed Muhammad Hannan A Rizvi, Joudi Sharaf, Kerry-Ann D Williams, Maha Tariq, Prachi Balani

**Affiliations:** 1 Internal Medicine, California Institute of Behavioral Neurosciences & Psychology, Fairfield, USA; 2 Ophthalmology, California Institute of Behavioral Neurosciences & Psychology, Fairfield, USA; 3 Research, California Institute of Behavioral Neurosciences & Psychology, Fairfield, USA; 4 Family Medicine/Dermatology, California Institute of Behavioral Neurosciences & Psychology, Fairfield, USA; 5 Medicine and Surgery, California Institute of Behavioral Neurosciences & Psychology, Fairfield, USA; 6 Neurology, California Institute of Behavioral Neurosciences & Psychology, Fairfield, USA; 7 Anesthesiology, California Institute of Behavioral Neurosciences & Psychology, Fairfield, USA; 8 Family Medicine, California Institute of Behavioral Neurosciences & Psychology, Fairfield, USA; 9 Internal Medicine, Saint Vincent Hospital, Worcester, USA

**Keywords:** gut-brain axis, irritable bowel syndrome, gastrointestinal motility disorders, ssri, serotonin

## Abstract

Gastrointestinal motility disorders have been thought to occur due to an imbalance in the interaction of the gut-brain axis, which is regulated by serotonin. This recent discovery can be exploited to find newer therapeutic agents such as selective serotonin reuptake inhibitors for functional gastrointestinal disorders.

PubMed, PubMed Central (PMC), and Medline databases were used to obtain the data. Meta-analyses, systematic reviews, randomized control trials, and reviews were included and analyzed in the data.

Of the 19240 studies, 23 were extracted, and after appropriate quality assessment, they were utilized in this systematic review. They included two meta-analyses, four systematic reviews, two randomized control trials, and 15 review articles. The systematic review focuses on the efficacy of selective serotonin reuptake inhibitors (SSRIs) as compared to other treatment modalities for disorders of gut-brain interaction. It explores various studies analyzing SSRIs for their mechanism of action, their desirable effects for treating irritable bowel syndrome, and their tolerability in patients.

SSRIs are effective and safe in treating overall symptoms of gastrointestinal motility disorders, particularly constipation-predominant disorders. They seem to have a better side effect profile than other drugs. This should encourage physicians to prescribe SSRIs early on in the disease.

## Introduction and background

Gastrointestinal motility disorders (previously called functional GI disorders) comprise majorly irritable bowel syndrome (IBS), seen in 9%-23% of the world population, and functional dyspepsia [1]. Globally, cases of gastrointestinal motility disorders have been rising for a long time. Even today, this ever-increasing burden of GI motility disorders has been taking a toll on the quality of life and available medical resources. The life of these patients is affected in many ways. They are constantly worried about eating habits, bloating, emotional stress due to altered bowel habits, consuming medications, absenteeism at work, and lower quality of life as compared to their peers [2].

IBS is a centerpiece of functional GI disorders [3]. Rome IV criteria are widely used for diagnosing the subtypes of IBS. The most common clinical features seen are altered bowel habits, changes in stool frequency and consistency, abdominal pain, and bloating [4]. Symptoms are usually chronic and relapsing in nature. The pathophysiology of these disorders is thought to be multifactorial. Disturbed gut motility, visceral hypersensitivity, gut mucosal immunity, altered gut microbiome, and disrupted central nervous system (CNS) processing that plays a role in the pathogenesis of these disorders [5]. Brain-gut axis has been a newer topic of interest in the pathophysiology of these disorders. Various neurotransmitters, including norepinephrine (NE), epinephrine (E), dopamine (DA), and serotonin (5-HT), affect the functioning of the GI tract [6]. The presence of serotonin reuptake transporters (SERT) in the gut wall proves that serotonin is a significant factor in gut peristalsis and maintaining the enteral neural system (ENS). There is also evidence of different types of serotonin receptors present on the gut wall. Serotonin increases gut motility, and its altered levels are partially responsible for gut inflammation [7]. Hence serotonergic drugs have become popular in managing delayed GI motility. Known and practiced treatment approaches for GI motility disorders are lifestyle modification, dietary changes, complementary and alternative medications, psychological and behavioral therapies, and pharmacotherapies [8]. Drugs used in the management of GI motility disorders are antispasmodics, intestinal secretagogues, antidiarrheals, laxatives, opioid agonists, probiotics, and for refractory or severe cases, antidepressants [9]. Tricyclic antidepressants (TCA) and selective serotonin reuptake inhibitors (SSRI) have shown clinical evidence of improvement of symptoms in constipation-predominant IBS (IBS-C) [10]. The role of serotonin in gut physiology is the basis of these treatments. SSRIs have long been used for the treatment of depression, but their implication in treating IBS-C is a crucial discovery.

Even after years of known and redefined pathophysiologies as well as advancements in pharmacotherapies for GI motility disorders, there is no specific cure. Only symptomatic management options are available. To increase the evidence for the use of current treatment choices, we need clearer and more detailed information on newer pharmacologic agents.

This study is a systematic review of the efficacy and safety of SSRIs in the management of GI motility disorders. This will help get more accurate and extensive clinical evidence on the role of SSRIs in treating IBS-C and delayed gut motility disorders.

Methods

A systematic review was conducted with the aid of Preferred Reporting Items for Systematic Review and Meta-Analyses (PRISMA) guidelines [11]. An initial, comprehensive search was done on April 24, 2022. Three databases were searched: PubMed, PubMed Central (PMC), and Medline. The basic search was carried out using the following concepts: (“selective serotonin reuptake inhibitors (SSRI)” or “antidepressants” or “serotonin”) AND (“gastrointestinal motility disorders” or “ irritable bowel syndrome” or “ functional gastrointestinal (GI) motility”). This yielded 811 studies. For a more targeted Medical Subject Heading (MeSH) search, SSRI medications were also added: fluoxetine OR paroxetine OR escitalopram OR citalopram OR sertraline. The final studies obtained were 19240. Table 1 explains the detailed search strategy.

**Table 1:** Search strategy SSRI - selective serotonin reuptake inhibitor, PMC - Pubmed Central, GI- gastrointestinal, MeSH- Medical Subject Heading

Search strategy	Databases	Number of studies (N=19240)
SSRI and GI motility disorders	PubMed, PMC, Medline	37
Antidepressants and GI motility disorders	PubMed, PMC, Medline	195
Escitalopram and GI motility disorders	PubMed, PMC, Medline	3
Paroxetine and GI motility disorders	PubMed, PMC, Medline	8
Fluoxetine and GI motility disorders	PubMed, PMC, Medline	8
Serotonin and GI motility disorders	PubMed, PMC, Medline	560
Combined terms in MeSH	PubMed, PMC, Medline	18429

Inclusion criteria

Before screening the studies, inclusion criteria were applied. Those with full free text in English and between the years 2013 and 2022 were considered. Only meta-analysis, systematic reviews, randomized controlled trials (RCT), and reviews were retained. Studies were limited to humans only. 

Exclusion criteria

Articles in other languages, before 2013, gray literature, and animal or clinical trials were excluded.

**Figure 1:**
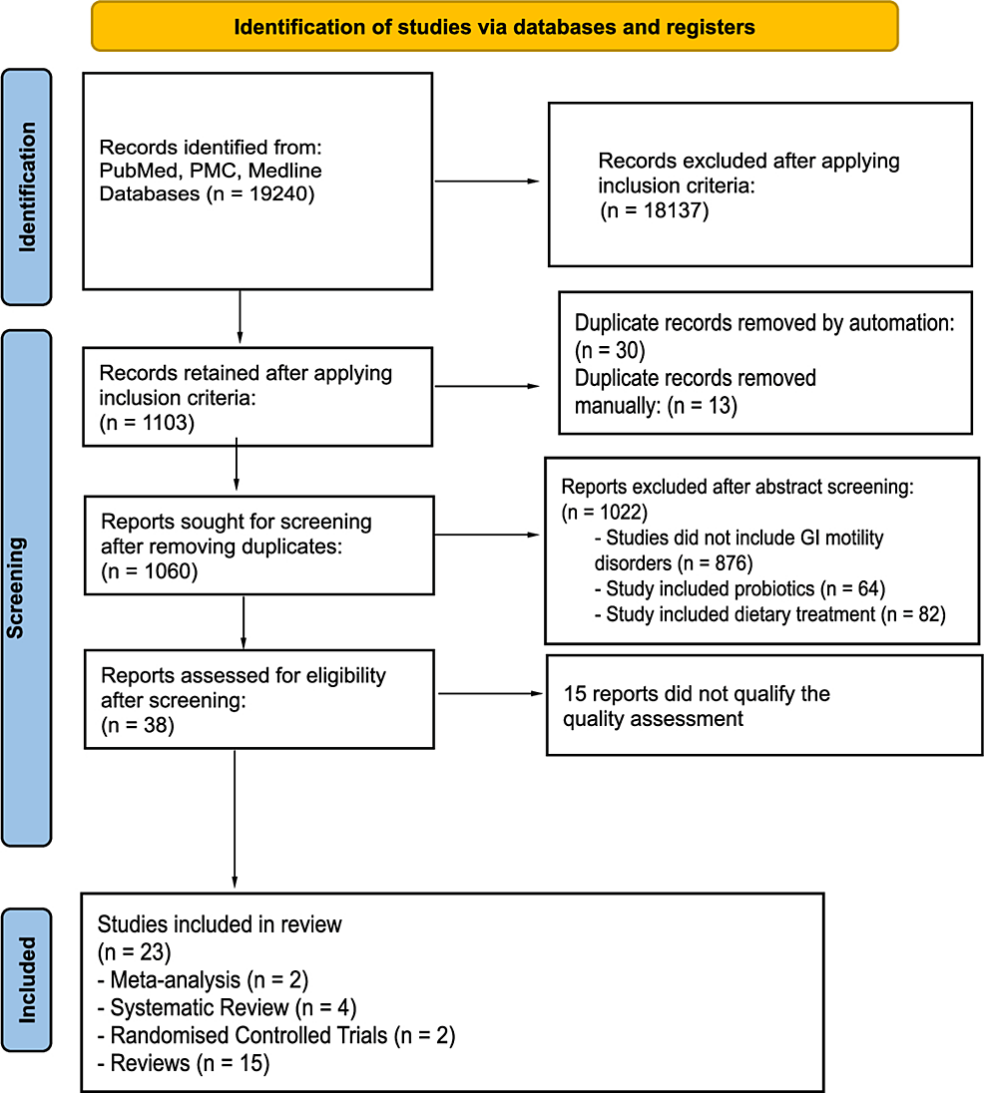
PRISMA flowchart 2020 - data extraction process PMC - PubMed Central, GI - gastrointestinal

Figure 1 explains the complete and sequential data extraction process [12]. 

Results

Table 1 and Figure 1 explain the search strategy and data extraction process respectively. Upon applying the search strategy, 19240 studies were obtained. The inclusion criteria were used, and duplicates were removed both by automation and manual methods. Those were then subjected to quality assessment using appropriate tools. After screening, a total of 23 studies were retained. The risk of bias in individual studies was reduced by using quality assessment tools: PRISMA for meta-analysis and systematic review; Cochrane Risk-of-bias tool for RCT; Scale for Assessment of Narrative Review Articles (SANRA) for review studies. 

They included two RCTs including 306 patients with an odds ratio of 0.94 in those administered escitalopram and p<0.05 [13,14]; four systematic reviews each studying six to 15 RCTs with greater significance and low risk of bias [9,15-17] and two meta-analyses [5,10]. One of which included seven RCTs with 346 patients comparing SSRI treatment with placebo treatment and showed that 54.5% (96/176) patients with SSRI trial demonstrated improvement in their symptoms (I²=49%, p=0.07) [5]. The other one included six RCTs with a low risk of bias and modest heterogeneity [10]. 

## Review

This systematic review aims to review trials of SSRIs in patients with Gastrointestinal motility disorders and find strong evidence of safety for their use.

GI motility disorders

GI motility disorders, or a more updated term disorders of gut-brain interaction (DGBI), include irritable bowel syndrome (majorly), functional dyspepsia, and functional constipation. IBS is classified into four different types according to Rome IV criteria: IBS-D (diarrhea-predominant), IBS-C (constipation-predominant), IBS-M (mixed- both diarrhea and constipation), and IBS-U (unclassified) [4]. Adriani et al. have described the universal Rome IV criteria for diagnosing IBS (Table 2) [4].

**Table 2:** Rome IV diagnostic criteria for irritable bowel syndrome Source: Adriani et al. [4]

Rome IV criteria
Recurrent abdominal pain, on average at least 1 day/week in the past 3 months and the onset of symptoms 6 months before diagnosing along with ≥2 of the following:
-Related to defecation
-Associated with a change in stool frequency
-Associated with a change in stool consistency (form).

Saha, in her review, highlights that the pathophysiology of IBS has been linked to changes in gastrointestinal motility, visceral hypersensitivity, post-infectious reactivity, brain-gut connections, fecal microflora, bacterial growth, food sensitivity, carbohydrate malabsorption, and intestinal inflammation [1]. We can say that targeting these aspects for management will lead to optimum remission of the disease. Radovanovic-Dinic et al. explain the minor role of genetic polymorphism in inflammatory factors such as catecholamine receptors, SERT, and cholecystokinin (CCK) receptors in determining the severity of symptoms [2]. This will help in the early diagnosis of DGBI in those with polymorphism and who receive targeted pharmacotherapy. Table 3 highlights the studies supporting data on GI motility disorders.

**Table 3:** Data on GI motility disorders IBS - irritable bowel syndrome, GI - gastrointestinal

Study	Author/ Reference no.	Year of Publication	Type of study	Purpose of study	Results	Interpretation
Irritable bowel syndrome subtypes: new names for old medical conditions	Grad et al. [3]	2020	Review	Review of new terminologies in IBS	The definition and Rome IV diagnostic criteria for IBS are mentioned	The article helps to diagnose the IBS patients
Irritable bowel syndrome - from etiopathogenesis to therapy	Radovanovic-Dinic et al. [2]	2018	Review	To summarize the etiology, pathogenesis, diagnostic criteria, and therapy for Functional GI disorders	The role of various neurotransmitters can be seen in the pathogenesis of IBS	Drugs modulating these neurotransmitters may be effective in the treatment of IBS
Irritable bowel syndrome: the clinical approach	Adriani et al. [4]	2018	Review	To explain updated views on various aspects of IBS	Mentions the major role of serotonin in the Gut-brain axis and the use of serotonergic drugs for the treatment of IBS	Pharmacotherapy targetting serotonin does seem to have benefit in IBS
Irritable bowel syndrome: pathogenesis, diagnosis, treatment, and evidence-based medicine	Saha [1]	2014	Review	Overview of pathogenesis, diagnosis, and treatment of IBS	This article highlights the pathophysiology of IBS including the role of serotonin, gut immunity, brain-gut interaction	The etiology of functional GI disorders is multifactorial

Serotonin

**Figure 2:**
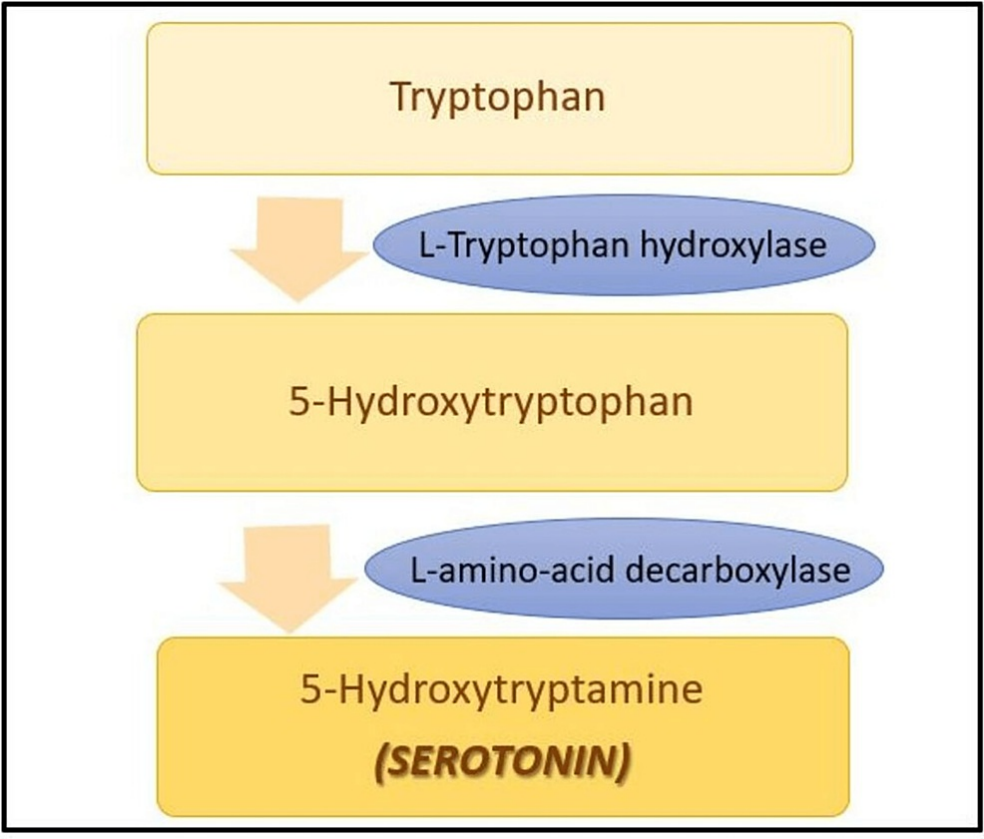
Biosynthesis of serotonin

Serotonin (5-hydroxytryptamine or 5-HT) is a key regulator of the gut-brain axis, and >90% of it is found in the GI tract. It is derived from tryptophan, an essential amino acid. In the GI tract, serotonin is produced by enterochromaffin cells (EC) and serotonergic neurons of the enteric nervous system (ENS), while in the CNS, it is produced only by serotonergic neurons. The biosynthesis of serotonin is shown in Figure 2.

Guzel et al. and Mittal et al. have mentioned seven types of serotonin receptors from 5-HT1-5-HT7 [6,7]. All receptors are G-protein coupled except 5-HT3 which is a ligand-gated ion channel receptor. The variety in receptors depicts extensive functions in various parts of the body. Their review also highlights the functions of each receptor. The functions have been summarized in Table 4 [7].

**Table 4:** Types of serotonin receptors and their functions 5-HT - 5-hydroxytryptamine, CNS - central nervous system, GI - gastrointestinal

Types of 5-HT receptors	Functions
5-HT_1a_	Gastric relaxation
5-HT_1b_	Initiation of peristalsis, contraction of the longitudinal muscle layer
5-HT_1d_	Contraction of the circular muscle layer
5-HT_1p_	Reflexes- secretory and peristaltic
5-HT_2a_	Contraction of smooth muscles
5-HT_2b_	Contraction of the gastric fundus, relaxation of intestinal longitudinal muscles
5-HT_3_	Secretion of serotonin from enterochromaffin cells, augments gut motility
5-HT_4_	Increased intestinal motility, relaxation of colon, secretion of chloride
5-HT_5_	Found only in CNS
5-HT_6_	Found only in CNS
5-HT_7_	Anti-inflammatory function in GI tract

The knowledge of varied functions carried out by serotonin in the gut will aid in prescribing a particular drug that acts at a specific choice of receptor. This will prevent unnecessary serotonin signaling and thus reduce adverse effects. Table 5 highlights the studies providing data on serotonin.

**Table 5:** Data on serotonin GI - gastrointestinal, IBS - irritable bowel syndrome

Study	Author/ reference no.	Year of publication	Type of study	Purpose of study	Results	Interpretation
The role of serotonin neurotransmission in gastrointestinal tract and pharmacotherapy molecules	Guzel et al. [7]	2022	Review	Identifying the role of serotonin in gut physiology and extrapolating its use to treat functional GI disorders.	Explain the role of serotonin in the gut-brain axis and IBS pathogenesis. Diarrhea-predominant IBS patients have high plasma serotonin levels and those with constipation-predominant IBS have low levels.	Serotonin agonists and antagonists and be used in IBS patients depending on their symptoms
Neurotransmitters: the critical modulators regulating gut-brain axis	Mittal et al. [6]	2017	Review	Illustrates the role of various neurotransmitters in gut physiology	Serotonin is a key regulator of gut immunity, motility, and inflammatory responses.	Serotonergic antagonists and agonists can be encouraged for the management of disorders of the gut-brain axis.

Gut-brain axis

The gut-brain-microbiota axis links the psychological and neurological system to the intestine, its occupants, and metabolic, neurohormonal, and immunologic activities. Moser et al. also explained that bacteria in the GI tract do affect the metabolism of the key regulator serotonin. Due to this reason, infections in the gut are responsible for the psychological symptoms in disorders of gut-brain interaction [18]. Mishima et al. published data saying altered gut flora causes fluctuations in the local serotonin levels, leading to changes in GI motility [19]. These altered serotonin levels in the gut are regulated by the serotonin reuptake transporter (SERT). This observation can be extrapolated for the use of pharmacologic agents acting on SERT, such as SSRIs in GI motility disorders. 

**Figure 3:**
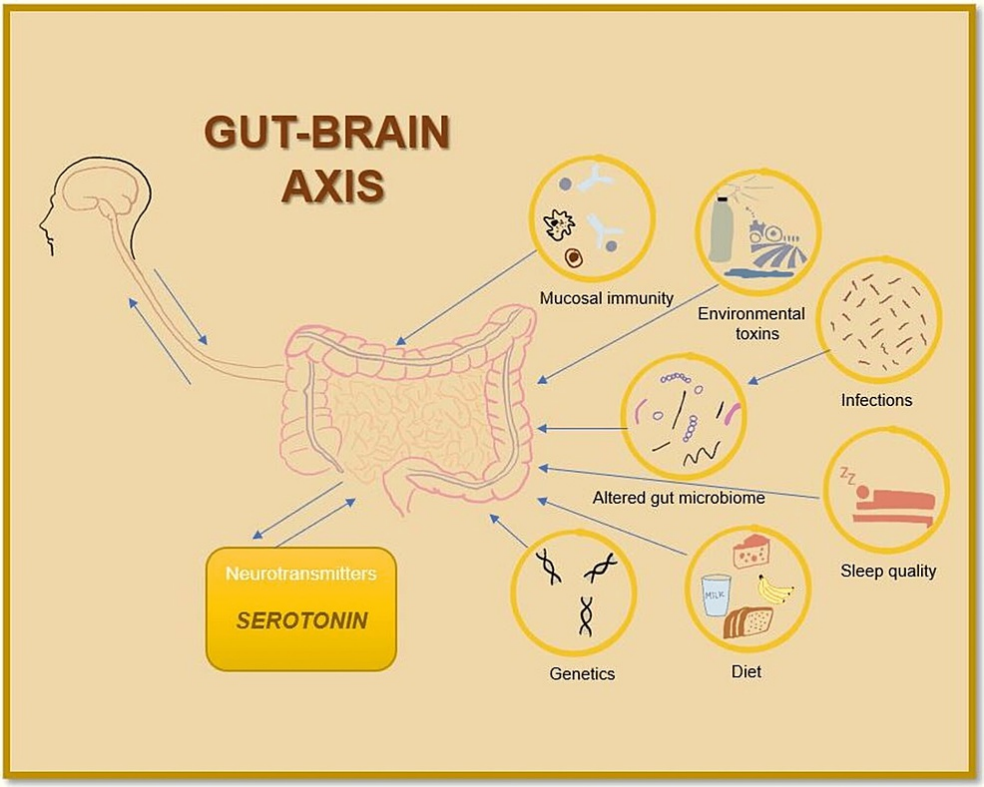
Gut-brain axis Illustration by author

The above two studies prove that changes in gut physiology result in nervous system symptoms, while conversely also holds. To support this, Aziz et al. stated in their review article that psychosomatic disorders, including depression, have two times greater chances of exhibiting GI symptoms of IBS [20]. This explains the bi-directional pathway of the gut-brain axis. Therefore the drug therapies used for psychosomatic disorders must be effective in DGBI, including antidepressants. To further support this, they also mention a high-quality RCT of vortioxetine (SSRI) by Seddighnia et al. involving 72 patients (36=vortioxetine, 36=placebo, p<0.01). Those who received vortioxetine demonstrated an increased quality of life with improvement in IBS symptoms like abdominal pain, stool consistency, and GI motility. Figure 3 shows the factors contributing to gut-brain axis.

Ghaffari et al., along with data depicting a link between the gut microbiome and the pathophysiology of GI motility disorders, also published data that demonstrates the use of SSRIs in IBS-C relieves pain as they help reduce the intestinal transit time [21]. The use of SSRIs for IBS-C cases should be promoted. Table 6 highlights the studies providing data on the gut-brain axis.

**Table 6:** Data on the gut-brain axis IBS - irritable bowel syndrome, SSRI - selective serotonin reuptake inhibitor, GI - gastrointestinal, CNS - central nervous system, SERT - serotonin reuptake transporters

Study	Author/ reference no.	Year of publication	Type of study	Purpose of study	Results	Interpretation
Irritable bowel syndrome and microbiome; switching from conventional diagnosis and therapies to personalized interventions	Ghafari et al. [21]	2022	Review	Applying computational modeling and machine learning to develop personalized treatment for IBS	Highlights the use of SSRIs for relieving pain in IBS patients as they decrease the transit time through the gut. Demonstrates connection between the gut microbiome and pathophysiology of functional GI disorders	Combined use of standard treatments of IBS with personalized intervention with newly emerging technology will pave the way for cure or at least prolonged remission of symptoms irritable
Irritable bowel syndrome, depression, and neurodegeneration: a bidirectional communication from gut to brain nutrients	Aziz et al. [20]	2021	Review	To discuss the communication of the enteric system and CNS	SSRIs when compared to placebo showed equivocal response in IBS patients	Since the trials show high heterogeneity the results need to be interpreted carefully
Enteric microbiota-mediated serotonergic signaling in pathogenesis of irritable bowel syndrome	Mishima et al. [19]	2021	Review	To describe recent advancements in the gut microbiome and serotonin connection	Microbial dysbiosis in the GI tract causes serotonin levels to alter the gut motility. Serotonin reuptake transporters (SERT) regulate the local serotonin levels in the gut as well as CNS	Drugs acting on SERT can change the serotonin levels in the gut and help alter the GI motility
Intestinal microbiome-gut-brain axis and irritable bowel syndrome	Moser et al. [18]	2018	Review	to establish a solid link between the intestinal microbiome and psychological features of IBS	Gut infections can contribute to psychological symptoms of functional GI disorders	The other perspective of psychological disorders causing GI symptoms also holds. Thus the pharmacological therapies used in these disorders may help in reducing GI symptoms

Mechanism of action of SSRIs

Abdominal Pain

As the name suggests, SSRIs selectively inhibit the serotonin reuptake into the cells (pre-synaptic in case of CNS action and EC cells in case of GI tract) by blocking SERT. This results in a sudden rise of serotonin levels either in the brain or gut and causes desired therapeutic effect. Camilleri, in his review, agrees that antidepressants have multifactorial effects in the treatment of DGBI [8]. The central (CNS) action is by decreasing the activation of pain centers in the brain in the anterior cingulate cortex and also reducing the central processing of pain stimuli, whereas the peripheral (GI tract) action is on alleviating pain by downregulating gut nociceptors, increasing intestinal compliance, and decreasing visceral afferents. This helps us understand the mechanism of action of SSRIs in conditions of chronic pain.

GI Motility

Along with improving abdominal pain in IBS, antidepressants also have effects on GI motility. TCAs are effective in IBS-D as they slow gut transit, and SSRIs are used for IBS-C as they hasten the transit [16].

Mawe et al., while explaining the function of SERT, say that serotonin transporters are present on epithelial cells in the intestine [22]. They reuptake the 5-HT molecule as soon as they are secreted by EC and thus modulate the levels of 5-HT in the mucosa. They also provide evidence for elevated serotonin levels in IBS-D and post-infectious-IBS, whereas reduced levels in IBS-C. It can be interpreted that the pharmacological blockade of SERT by SSRI can increase the local levels of serotonin and relieve the symptoms of IBS-C. Table 7 highlights the studies providing data on the mechanism of action of SSRIs.

**Table 7:** Data on the mechanism of action of SSRIs GI - gastrointestinal, SERT - serotonin reuptake transporter, IBS - irritable bowel syndrome, IBS-C -​​​​​ irritable bowel syndrome​​​​​​ constipation-predominant, IBS-D - irritable bowel syndrome diarrhea-predominant

Study	Author/ Reference no.	Year of Publication	Type of study	Purpose of study	Results	Interpretation
Management options for irritable bowel syndrome	Camilleri [8]	2018	Review	Summarizes the mechanism of action, efficacy, and safety of each intervention for IBS	It demonstrates the link between central pain perception, altered motility, secretions of the GI tract, and altered sensations in the gut mucosa	This explains the rationale for using a particular drug for a specific type of IBS
Serotonin signalling in the gut--functions, dysfunctions and therapeutic targets	Mawe et al. [22]	2013	Review	Outlines the functions of serotonin and its use in GI pharmacology.	Levels of serotonin are elevated in cases of IBS-D and post-infectious IBS whereas they are reduced in IBS-C	These observed changes in the plasma serotonin levels can be exploited to develop more targeted therapies for IBS

Efficacy

In a high-quality RCT (2021), performed by Kreiter. et al., a total of 14 IBS patients were grouped into placebo (n=9) and escitalopram (n=5). Results were obtained using node strength and were statistically significant. They showed a positive effect on the improvement of both physical and mood symptoms [14]. On comparing these results with another RCT (2015) by Talley et al. that included 292 subjects with functional dyspepsia assigned to three groups, placebo (n=97), amitriptyline (n=97), and escitalopram (n=98) [13], it showed that relief rates were higher in the amitriptyline group (53%) as opposed to 40% with placebo and 38% with escitalopram. Of the three groups, none was able to relieve gastric emptying or meal-related satiety significantly. However, those with delayed gastric emptying did exhibit relief in the escitalopram group as compared to the placebo. It can be said that if the subject groups were to be classified according to the symptom predominance, there would have been more conclusive data for the use of a specific drug in a certain type of disorder.

A systematic review by Qin et al. included 12 studies on antidepressants, including 948 patients of all types of IBS [9]. The risk ratio (RR) for overall symptom relief was 1.56 with a 95% confidence interval (CI) (1.19-2.04) and p<0.05. In another systematic review by Black et al., which included 10 RCTs on TCAs and five RCTs on SSRIs, TCAs (RR=0.66, 95%CI 0.53-0.83, p=0.77) showed greater benefit over SSRIs [15]. While both studies support the use of antidepressants, when choosing the type of antidepressants, TCAs seem to be most likely prescribed over SSRIs. This could be because the trials with SSRIs are lower in number, include a lesser subject population, and have higher heterogeneity.

Gewandter et al., in their systematic review of chronic pain conditions, highlight the relief of abdominal pain in IBS when pharmacologic therapy is utilized [16]. To support this, they have included the results of six RCTs for SSRI along with other pharmacologic options used for IBS. This study only includes pain symptoms and cannot provide conclusive evidence for the relief of global symptoms of IBS. Scaciota et al. published data emphasizing the efficacy of antidepressants (TCAs and SSRIs) in IBS in their systematic review [17]. They studied 15 RCTs, of which 11 RCTs with 750 subjects were interpreted for an overall assessment of relief, and the results were RR=1.57, 95% CI=1.23-2.0. This study does not differentiate between different drug classes used in the RCT; therefore, the exact efficacy of SSRIs is difficult to interpret.

A meta-analysis by Ford et al. included 18 RCTs (11=TCAs, 6=SSRIs, 1=both) for antidepressants with 1127 patients compared either against placebo or psychological therapy [5]. The results specific for SSRIs vs placebo (356 patients) those not showing improvement, RR=0.68, 95%CI=0.51-0.91, p=0.07, and heterogeneity (I)=49%. 54.5% of patients assigned to the SSRI group showed improvement as opposed to 32.8% in the placebo. It can be summarized that the greater efficacy of SSRIs can be used as evidence in the management of IBS. A meta-analysis conducted in 2015 by Xie et al. incorporated six trials for SSRIs which had a total of 371 subjects, and the results procured showed improved symptoms of IBS with RR=1.38, 95%CI=0.83-2.28, p=0.01% and I=57% [10]. When compared to TCAs, trials with SSRIs have higher heterogeneity and, therefore, are less likely to provide strong evidence for their use. The meta-analysis also mentions that some studies of SSRIs that included only IBS-C patients and treated with fluoxetine (SSRI) were found to have reduced abdominal discomfort, increased stool frequency, and better stool consistency [10]. This concludes that SSRIs can be effectively used in patients with IBS-C but with lesser efficacy in other types of IBS. 

While the above studies help understand the efficacy of SSRIs for GI symptoms of IBS, a review by Törnblom et al. highlights that SSRIs also alleviate the accompanying mood symptoms, including anxiety, depression, and psychological distress [23]. Thus they improve the quality of life in patients with co-morbid psychiatric disorders. Table 8 highlights the studies providing data on the efficacy of SSRIs.

**Table 8:** Data on the efficacy of SSRIs RCT - randomized controlled trial, IBS - irritable bowel syndrome, SSRI - selective serotonin reuptake inhibitor, TCA - tricyclic antidepressant, GI - gastrointestinal

Study	Author/ reference no.	Year of publication	Type of study	No. of patients	Purpose of study	Results	Interpretation
Symptom-network dynamics in irritable bowel syndrome with co-morbid panic disorder using electronic momentary assessment: a randomized controlled trial of escitalopram vs. placebo	Kreiter et al. [13]	2021	RCT	14	To establish the effectiveness of Escitalopram in IBS patients with underlying co-morbid panic disorders	Use of escitalopram in IBS patients showed improvement in physical symptoms such as abdominal pain, bloating, and nausea as compared to placebo. Also described enhancement in mood including being more happier and less anxious and lonely	SSRIs when used in IBS patients aid in improving their quality of life by relieving physical as well as mood symptoms
Interventions for the treatment of irritable bowel syndrome: a review of Cochrane systematic reviews	Scaciota et al. [17]	2021	Systematic review	750	To know the efficacy of various interventions for IBS	Pharmacological treatment with antidepressants including TCAs and SSRIs presented with clinical improvement	Pharmacological treatments seem superior to nonpharmacologic treatment except for psychologic therapy which has similar efficacy in IBS
Pharmacologic treatments for irritable bowel syndrome: an umbrella systematic review	Qin et al. [9]	2020	Systematic review	948	To strengthen the evidence of pharmacological treatments for IBS and aid in standardizing the therapy for IBS	Those treated with pharmacological therapy show improvement in major IBS symptoms such as abdominal pain, bloating, defecation urgency	This further encourages the use of drugs for the treatment of IBS or at least in refractory cases
Efficacy of soluble fibre, antispasmodic drugs, and gut-brain neuromodulators in irritable bowel syndrome: a systematic review and network meta-analysis	Black et al. [15]	2020	Systematic review	-	Comparing and ranking the traditional therapies for functional GI disorders according to their efficacy	TCAs showed greater efficacy than any other treatment for IBS but also greater adverse effects	SSRIs can be preferred over TCAs to avoid the adverse effects of TCAs but conclusive evidence is still needed
Effect of antidepressants and psychological therapies in irritable bowel syndrome: an updated systematic review and meta-analysis	Ford et al. [5]	2019	Meta-analysis	356	To explore the role of centrally acting drugs in the management of Functional GI disorders	SSRIs as compared to TCAs showed lower efficacy in alleviating the symptoms but at the same time have lower side effects	The use of SSRI does not hold strong evidence since the trials conducted have higher heterogeneity
Research design characteristics of published pharmacologic randomized clinical trials for irritable bowel syndrome and chronic pelvic pain conditions: an ACTTION systematic review	Gewandter et al. [16]	2018	Systematic review	-	Interprets the results of high-quality RCTs and evaluates the clarity of analyses proposed by those studies.	This systematic review shares evidence for drug therapy for chronic pain conditions including abdominal pain for IBS	The focus of treating IBS should be on relieving most of the symptoms of IBS and enhancing their quality of life and not only on alleviating abdominal pain
Psychotropics, antidepressants, and visceral analgesics in functional gastrointestinal disorders	Törnblom et al. [23]	2018	Review	-	Provides updated evidence on the use of neuromodulators such as antidepressants, antianxiety, and antipsychotic medications in IBS	The basis for clinical use of neuromodulators in IBS is well established.	The efficacy of these drugs in treating functional GI disorders proves the role of the gut-brain axis in its etiology.
Efficacy and safety of antidepressants for the treatment of irritable bowel syndrome: a meta-analysis	Xie et al. [10]	2015	Meta-analysis	371	Evaluate the use of antidepressants in treating IBS	TCAs demonstrate holistic improvement in symptoms of IBS. For encouraging the prescription of SSRIs substantial evidence is needed.	There is a need for more trials on SSRIs as they are more efficacious and less adverse events when used. The lack of trials is an obstacle to the standardized use of SSRIs.
Effect of amitriptyline and escitalopram on functional dyspepsia: a multicenter, randomized controlled study	Talley et al. [14]	2015	RCT	292	Comparison between amitryptiline and escitalopram for their efficacy and safety in functional GI disorders	Amitryptiline showed greater relief than escitalopram but none showed any efficacy in improving the gastric emptying and meal-induced satiety symptoms of IBS. Both were concluded to be useful management options in IBS.	Heterogenous results for amitryptiline and escitalopram infer that treatment must be individualized. Between TCAs and SSRIs, the physician should make an appropriate choice suitable for the patient depending on the patient's response.

Adverse effects

The common adverse effects noted by Xie et al. in their study were headache, poor sleep, anxiety, and nausea [10]. Although SSRIs show lower efficacy than TCAs, they are more tolerable. They show significantly lower drop-out rates than TCAs. Qin et al. and Wall et al. both in their studies also reinforce the common side effects of SSRIs found were headache and nausea, but still demonstrated greater tolerability than TCAs [9,24]. The fact that SSRIs lack anticholinergic, cardiovascular, and neurological side-effects seen in TCAs makes them a preferred choice over TCAs. Table 9 highlights studies providing data on the adverse effects of SSRIs.

**Table 9:** Data on the adverse effects of SSRIs IBS - Irritable bowel syndrome, RR - relative risk, CI - confidence interval, TCA - tricyclic antidepressants, SSRI - selective serotonin reuptake inhibitors

Study	Author/ reference no.	Year of publication	Type of study	Purpose of study	Results	Interpretation
Pharmacologic treatments for irritable bowel syndrome: an umbrella systematic review	Qin et al. [9]	2020	Systematic review	To strengthen the evidence of pharmacological treatments for IBS and aid in standardizing the therapy for IBS	The overall adverse event rate for antidepressants (RR = 1.56, 95% CI: 1.23-1.98)	Even though antidepressants used were on low dose, side effects were seen
Efficacy and safety of antidepressants for the treatment of irritable bowel syndrome: a meta-analysis	Xie et al. [10]	2015	Meta-analysis	Evaluate the use of antidepressants in treating IBS	RR of drop-out rates for TCAs was 1.92 (95% CI: 0.89-4.17) and RR of drop-out rates for SSRIs was 1.5 (95% CI: 0.67-3.37)	Drop-out rates for SSRIs are lower than TCAs
Irritable bowel syndrome: a concise review of current treatment concepts	Wall et al. [24]	2014	Review	Charts the growth of treatment options from primitive drugs to newer agents	A trial with desipramine showed a lesser drop-out rate due to adverse effects	SSRIs class of drugs demonstrate greater tolerability

Limitations

This systematic review has certain limitations, such as few studies included had a lesser population indicating lower power of the study. Also, those studies with no or low statistical significance would be unavailable or unpublished and therefore were unable to procure them. Studies in any other language than English were not included in this review.

## Conclusions

SSRIs are quite efficient and safe in improving the global symptoms of Gastrointestinal motility disorders or DGBI, especially in cases of IBS-C. Early diagnosis and appropriately classifying into IBS subtypes are crucial in deciding the pharmacological therapy for DGBI. Serotonin-targeted therapies are key in managing IBS, mainly in those with abnormal SERT polymorphism. SSRIs have shown promising effects in cases of delayed intestinal motility symptoms of IBS. SSRIs have demonstrated central as well as peripheral therapeutic effects and a better side-effect profile than TCAs.

In our opinion, larger trials of SSRIs specific for IBS-C are lacking. This will encourage physicians to prescribe SSRIs in cases of constipation-predominant symptoms. Furthermore, even after decades of diagnosing these disorders, no cure has been discovered. With the increasing incidence of DGBI, there may be ways to prevent the occurrence, such as dietary habits, lifestyle changes, and early stress management strategies.

## References

[1] Saha L (2014). Irritable bowel syndrome: pathogenesis, diagnosis, treatment, and evidence-based medicine. World J Gastroenterol.

[2] Radovanovic-Dinic B, Tesic-Rajkovic S, Grgov S, Petrovic G, Zivkovic V (2018). Irritable bowel syndrome - from etiopathogenesis to therapy. Biomed Pap Med Fac Univ Palacky Olomouc Czech Repub.

[3] Grad S, Dumitrascu DL (2020). Irritable bowel syndrome subtypes: new names for old medical conditions. Dig Dis.

[4] Adriani A, Ribaldone DG, Astegiano M, Durazzo M, Saracco GM, Pellicano R (2018). Irritable bowel syndrome: the clinical approach. Panminerva Med.

[5] Ford AC, Lacy BE, Harris LA, Quigley EM, Moayyedi P (2019). Effect of antidepressants and psychological therapies in irritable bowel syndrome: an updated systematic review and meta-analysis. Am J Gastroenterol.

[6] Mittal R, Debs LH, Patel AP (2017). Neurotransmitters: the critical modulators regulating gut-brain axis. J Cell Physiol.

[7] Guzel T, Mirowska-Guzel D (2022). The role of serotonin neurotransmission in gastrointestinal tract and pharmacotherapy. Molecules.

[8] Camilleri M (2018). Management options for irritable bowel syndrome. Mayo Clin Proc.

[9] Qin D, Yue L, Xue B, Chen M, Tang TC, Zheng H (2019). Pharmacological treatments for patients with irritable bowel syndrome: an umbrella review of systematic reviews and meta-analyses. Medicine (Baltimore).

[10] Xie C, Tang Y, Wang Y, Yu T, Wang Y, Jiang L, Lin L (2015). Efficacy and safety of antidepressants for the treatment of irritable bowel syndrome: a meta-analysis. PLoS One.

[11] Bellini M, Gambaccini D, Stasi C, Urbano MT, Marchi S, Usai-Satta P (2014). Irritable bowel syndrome: a disease still searching for pathogenesis, diagnosis and therapy. World J Gastroenterol.

[12] Page MJ, McKenzie JE, Bossuyt PM (2021). The PRISMA 2020 statement: an updated guideline for reporting systematic reviews. BMJ.

[13] Talley NJ, Locke GR, Saito YA (2015). Effect of amitriptyline and escitalopram on functional dyspepsia: a multicenter, randomized controlled study. Gastroenterology.

[14] Kreiter D, Drukker M, Mujagic Z (2021). Symptom-network dynamics in irritable bowel syndrome with comorbid panic disorder using electronic momentary assessment: a randomized controlled trial of escitalopram vs. placebo. J Psychosom Res.

[15] Black CJ, Yuan Y, Selinger CP, Camilleri M, Quigley EMM, Moayyedi P, Ford AC (2020). Efficacy of soluble fibre, antispasmodic drugs, and gut-brain neuromodulators in irritable bowel syndrome: a systematic review and network meta-analysis. Lancet Gastroenterol Hepatol.

[16] Gewandter JS, Chaudari J, Iwan KB (2018). Research design characteristics of published pharmacologic randomized clinical trials for irritable bowel syndrome and chronic pelvic pain conditions: an ACTTION systematic review. J Pain.

[17] Scaciota AC, Matos D, Rosa MM, Colovati ME, Bellotto EF, Martimbianco AL (2021). Interventions for the treatment of irritable bowel syndrome: a review of cochrane systematic reviews. Arq Gastroenterol.

[18] Moser G, Fournier C, Peter J (2018). Intestinal microbiome-gut-brain axis and irritable bowel syndrome. Wien Med Wochenschr.

[19] Mishima Y, Ishihara S (2021). Enteric microbiota-mediated serotonergic signaling in pathogenesis of irritable bowel syndrome. Int J Mol Sci.

[20] Aziz MNM, Kumar J, Muhammad Nawawi KN, Raja Ali RA, Mokhtar NM (2021). Irritable bowel syndrome, depression, and neurodegeneration: a bidirectional communication from gut to brain. Nutrients.

[21] Ghaffari P, Shoaie S, Nielsen LK (2022). Irritable bowel syndrome and microbiome; switching from conventional diagnosis and therapies to personalized interventions. J Transl Med.

[22] Mawe GM, Hoffman JM (2013). Serotonin signalling in the gut-functions, dysfunctions and therapeutic targets. Nat Rev Gastroenterol Hepatol.

[23] Törnblom H, Drossman DA (2018). Psychotropics, antidepressants, and visceral analgesics in functional gastrointestinal disorders. Curr Gastroenterol Rep.

[24] Wall GC, Bryant GA, Bottenberg MM, Maki ED, Miesner AR (2014). Irritable bowel syndrome: a concise review of current treatment concepts. World J Gastroenterol.

